# Toxicological evaluation of exhaust emissions from light-duty vehicles using different fuel alternatives in sub-freezing conditions

**DOI:** 10.1186/s12989-020-00348-0

**Published:** 2020-05-27

**Authors:** Henri Hakkarainen, Päivi Aakko-Saksa, Maija Sainio, Tuukka Ihantola, Teemu J. Rönkkö, Päivi Koponen, Topi Rönkkö, Pasi I. Jalava

**Affiliations:** 1 Inhalation toxicology laboratory, Department of Environmental and Biological Sciences, University of Eastern Finland, P.O. Box 1627, 70211 Kuopio, Finland; 2 VTT Technical Research Centre of Finland, VTT, P.O. Box 1000, 02044 Espoo, Finland; 3 Aerosol Physics Laboratory, Physics Unit, Tampere University, P.O. Box 692, 33014 Tampere, Finland

**Keywords:** Diesel, Gasoline, Compressed natural gas, Particulate matter, Emissions, In vitro toxicology

## Abstract

**Background:**

Emissions from road traffic are under constant discussion since they pose a major threat to human health despite the increasingly strict emission targets and regulations. Although the new passenger car regulations have been very effective in reducing the particulate matter (PM) emissions, the aged car fleet in some EU countries remains a substantial source of PM emissions. Moreover, toxicity of PM emissions from multiple new types of bio-based fuels remain uncertain and different driving conditions such as the sub-zero running temperature has been shown to affect the emissions. Overall, the current literature and experimental knowledge on the toxicology of these PM emissions and conditions is scarce.

**Methods:**

In the present study, we show that exhaust gas PM from newly regulated passenger cars fueled by different fuels at sub-zero temperatures, induce toxicological responses in vitro. We used exhaust gas volume-based PM doses to give us better insight on the real-life exposure and included one older diesel car to estimate the effect of the new emissions regulations.

**Results:**

In cars compliant with the new regulations, gasoline (E10) displayed the highest PM concentrations and toxicological responses, while the higher ethanol blend (E85) resulted in slightly lower exhaust gas PM concentrations and notably lower toxicological responses in comparison. Engines powered by modern diesel and compressed natural gas (CNG) yielded the lowest PM concentrations and toxicological responses.

**Conclusions:**

The present study shows that toxicity of the exhaust gas PM varies depending on the fuels used. Additionally, concentration and toxicity of PM from an older diesel car were vastly higher, compared to contemporary vehicles, indicating the beneficial effects of the new emissions regulations.

## Introduction

During the past decade, European Union has aimed at lowering greenhouse gas emissions from road traffic, which in the first phase has led to substantial increase in share of more energy efficient diesel-powered vehicles in the fleet [4, 18, 74]. However, as diesel-powered vehicles are major emitters of nitrogen dioxide (NO_2_), this has led to increased emissions of NO_2_ in EU area [19]. Another intrinsic feature of a diesel engine has been its higher particulate matter (PM) mass emissions, compared to gasoline powered engines. These components of the vehicle emissions have crucial importance to the air quality, and thus, with addition of CO_2_, have been targeted in multiple emission controls decisions in Europe. Example of these emissions controls is the Euro emissions standards for traffic which started at 1992 in European area with aim to lower traffic-based emissions. The diesel particulate filters (DPF) were introduced with the Euro 5 emission standard to meet the stringent exhaust particle emission limits and have led to significant decrease in PM emissions from diesel cars. Moreover, NO_x_ emissions have been effectively regulated in the newest emission standards. However, air quality in Europe has not improved as much as it should have despite the increasingly strict emission limitations [20]. One of the major reasons for this is the aged car fleet, as the age of the fleet is getting older year on year with an average age of passenger cars being 11.1 years in 2017 [5], with a large variation between countries. In fact, the CO_2_ emissions from traffic have increased in the EU as well, in spite of the emissions targets. This is both due to, increased total number of vehicles [23], and due to a recent shift from diesel to gasoline powered cars. Overall, for the internal combustion engine powered fleet, finding the balance between different emissions and adverse health effects induced by the emissions is very challenging even without adding the alternative fuels into the equation.

The current discussion on traffic emissions and their effect to air quality has been largely focused to vehicles powered by diesel engines. However, recent findings show that modern gasoline powered engines produce at least equal emissions of nano-sized particles compared to the modern diesel engines [37, 45, 69]. Therefore, limiting only the diesel vehicle emissions due to NO_x_ without requirement of particle filters for gasoline cars emissions, may lead to increased emissions of the smallest particles, which could have large and unpredictable effects on the human health. Moreover, effects of alternative fuels, such as ethanol and natural gas to vehicle emissions, air quality, and health are studied insufficiently. So far studies have shown that compressed natural gas (CNG) fuelled cars equipped with three-way catalyst (TWC) result to very low particle number emissions [1], and PM emissions from CNG fuelled cars have been associated with lower concentrations of PM-bound carcinogenic PAHs, compared to gasoline and diesel cars [7]. Additionally, the higher ethanol blend in gasoline has been detected to result in lower PM emissions in exhaust gases with less toxicologically active PAH compounds [40, 71], which have also been observed at sub-freezing ambient temperatures [2, 54]. Hence, the running and starting conditions influence the emissions in addition to differences in fuels. For example, cold temperature is linked to higher emissions from passenger cars, affecting most notably PM and NO_x_ emissions [52, 67]. However, the effect of the cold temperature to toxicity of the emissions remains inadequately studied, although some studies have been conducted [2]. Furthermore, PM emissions are not taken into the account in EU cold temperature motor vehicle emissions test (The Type 6 test) which mitigates the relevance and comparability of the emissions test to real-life conditions [52].

The adverse health effects of traffic related aerosols have been shown in many recent publications. In the study of Samoli et al. [47] both diesel and gasoline vehicle exhaust were associated with hospital admissions in London, and in the study of Tobías et al. [55] traffic related ultrafine particles were connected to daily mortality in Spanish cities. Besides, in the study of Park et al. [41] PM from several different sources were evaluated by toxicity responses from multiple toxicological endpoints; PM originated from diesel and gasoline engines were the two with the most prominent toxicological capacity. This suggests that of air pollution components at large, traffic-originated PM induces the highest toxicological responses. This agrees with the International Agency for Research on Cancer (IARC) which has categorized diesel exhaust gas as carcinogenic to humans (Group 1) and gasoline exhaust to possibly carcinogenic to humans (Group 2B) [26].

The current regulations of air quality are based on particulate mass. However, with the reduced PM mass, the emissions from internal combustion engines stays still a potential threat to human health. Thus, alternative approaches are needed to assess the harmfulness of especially the modern technologies with small mass-based emissions. Indeed, the particle numbers have been included in the newest emission standards, but unfortunately in those, only particles > 23 nm are measured. Particle number (PN) may have significant adverse health effects though. There are also indications that total surface area of PM and elemental carbon (or black carbon) has adverse health effects and many of them could be associated to ultrafine particle emissions from traffic [6, 42].

In the present study RAW264.7 mouse macrophages were exposed to exhaust gas volume-based doses of PM samples from four different Euro 6a emission regulated cars and one Euro 2 emission regulated car. The doses of PM correspond to mass of PM from certain volumes of the diluted exhaust gas. However, as the PM mass concentrations were up to several folds higher from the Euro 2 car, to avoid pernicious cytotoxicity, the doses of PM for Euro 2 diesel car had to be one tenth of those used with Euro 6a cars originated PM. This exhaust gas volume-based exposure method includes differences in PM concentrations of exhaust gases from different cars, giving us better view of the real-life exposures and toxicity of the exhaust gas PM, caused by different types of passenger cars on the streets. After the exhaust gas PM exposures, toxicological responses were assessed by several endpoint analyses, including cytotoxicity, inflammatory mediators, oxidative stress and mutagenicity. Additionally, the chemical characterization of the collected PM was performed as described in detail by [3].

## Materials and methods

All the details about the engines, fuels, and sampling methods are elaborated in Technical Research Center of Finland (VTT) report of Aakko-Saksa et al. [3]. Some of the important factors are presented briefly in the chapters below.

### Cars and fuels

PM was collected from exhaust of four Euro 6a emission regulated cars and from one Euro 2 regulated car by the VTT. EN590 winter-grade diesel fuel was used in the Euro 2 diesel powered car (DI-E2) whereas regular EN590 diesel was used in the Euro 6a diesel car (DI-E6). Other Euro 6a cars used compressed natural gas (CNG), gasoline with 10% ethanol (E10) and high-blend ethanol fuel with 83/17% ethanol-gasoline-ratio (E85). The Euro 6a cars used turbocharged direct injection engines and the exhaust gas after-treatment-system were three-way catalyst (TWC) for E10, E85 and CNG -powered cars, and for DI-E6, NO_x_ adsorber catalyst, exhaust recirculation (EGR) systems and diesel particle filter (DPF). The Euro 2 diesel car had normal single injection diesel engine without any exhaust aftertreatment system. Detailed information of the engines and fuels is represented in the report [3].

### Sampling and measuring of the particles

PM sample collection was performed using chassis dynamometer on climatic test of − 7 °C under New European Driving Cycle (NEDC) according to UN ECE R83 regulation. The driving cycle was total of 11.0 km divided into three test phases. The first and second test phases each consisted of 2.026 km driving with urban driving cycle (ECE15), and the third test phase, extra-urban driving cycle (EUDC), was 6.955 km.

Measurement of PM emissions and collection of samples for PAH, micro-ames, and DTT analyses were conducted with high capacity collection system. System includes constant volume sampler (CVS i60 LD, Venturi-type, AVL, Austria), a dilution tunnel (Ø 265 mm, 400 cm, Pierburg, GmbH, Germany), a sample probe (Ø 80 mm), two filter holders in parallel (Ø 142 mm), a blower (Siemens ELMO-G, 2BH1 810-1HC36, 11 kW, Germany), a flow meter (Bronkhorst F106C1-HD-V-12, Netherlands) and controller (Stafsjö MV-E-80-P-TY-AC100-PN10, Sweden). Dilution of exhaust gas was carried in dilution tunnel at flow rates between 3 and 18 m^3^/min. The dilution of the exhaust gases varied between the cars; the volumes of the diluted exhaust gases per driving distance for each car were the following; DI-E2: 13.08 m^3^/km, CNG: 9.75 m^3^/km, E10: 9.68 m^3^/km, E85 9.70 m^3^/km, and DI-E6: 9.73 m^3^/km. The dilution rates (DR) and volumes of raw exhaust gases are presented in more detail from each car (Supplementary material table 1. Additional file 1).

After the dilution, the flow was adjusted to 200–1500 L/min and two Ø 142 mm polytetrafluoroethylene (PTFE) (Fluoropore 3.0 FSLW, Millipore, USA) filters were used in parallel. Followed by the sampling, the filters were weighed and then wrapped in aluminium foil to protect them from light. Particulate filters were then stored in freezer and sent for further analysis. The PM mass emissions were calculated based on the high-capacity collection system. Blank control filters were treated similarly as the actual PM sample filters with exception of sampling from the exhaust gas.

Measurements of the non-volatile PN were conducted in compliance with Euro 6 regulation (EU No 133/2014) by butanol Condensation Particle Counter (bCPC) (Airmodus A23, Finland). The bCPC was set to measure particles with the size range of 23 nm to 2.5 μm.

The preparation of the PM samples for toxicological analyses has been described in detail previously by [28, 29]. Briefly, the PTFE filters were cut into four pieces and placed in 50 ml glass tubes. Glass tubes were then filled with methanol, followed by extraction/suspension of samples by total of 2 × 30 min sonication in an ultrasonic water bath at a temperature below 35 °C. Thereafter, the suspensions were concentrated with a rotatory evaporator to a small volume and divided into glass tubes on calculated emission volume basis. Finally, these samples were dried in the glass tubes by flow of nitrogen gas (99.5%) and stored at − 20 °C.

### Chemical characterization and mass of the particles

PM associated components such as polyaromatic hydrocarbons (PAH) and different anions were analysed as described in report of [3]. Anions analysed were SO_4_^2−^, NO_3_^−^ and Cl^−^. Total of 24 PAH compounds were analysed from the PM according to ISO 16000-6:2011 and EN 14662–4:2005 analysis method. These 24 PAH compounds included PAHs from US EPA 16 [60] list, from which many compounds have been classified as carcinogenic. In addition to US EPA 16 list, the measured PAHs also included 6 priority PAHs from the list of mobile-source air toxics defined by US EPA [61]. Moreover, the mass of the collected PM was measured from the PTFE-filters after the sampling.

For the elemental carbon (EC) and organic carbon (OC) ratio (EC/OC) measurements, the sample collection was carried out with Ø 47 mm quartz filters (MK 360, Munktell, Sweden) which were heated in an oven at 700 °C for 1 h and stabilized prior sample collection. EC/OC samples were collected to quartz filters from DI-E2, E10, and E85 cars, using standard particle collection system. CNG and DI-E6 emissions contained such small amounts of carbon components that EC/OC ratios could not be measured from these small sample masses in the present study. Briefly, the PM were collected over the whole driving cycle to one filter for E10 and E85, while for DI-E2, the samples were collected in three phases due to its high PM emission level. Sample flow was adjusted to 17.5 L/min and once to 7.5 L/min for DI-E2 to obtain suitable darkness of samples for the EC/OC analysis.

EC/OC was analysed at VTT using thermo-optical EC/OC analyser (Internal method 25.01, Model 4 l, Sunset Laboratories Inc., USA). Transmission of laser is measured through the sample, while temperature and gas atmosphere are adjusted. Followed by the second phase, in which the sample is cooled, O_2_/He is introduced, and temperature is raised. Carbon is oxidized to CO_2_, which is then converted to methane and detected by the flame ionization detector (FID). The organic compounds pyrolytically converted to EC are compensated by continuous measurement of the transmission of a laser. Later, the quantities of OC and EC, with addition of total carbon (TC), in the sample were calculated based on the FID response and laser transmission data. Methane and saccharose were used for calibration.

### Oxidative potential of the particles

Oxidative potential of the collected particles were tested with the Dithiothreitol (DTT = HSCH_2_(CH (OH))_2_CH_2_SH) assay according to Charrier & Anastasio [13] with the exception that there was used 200 μM of DTT in Chelex which were treated with 100 μM phosphate buffer (77.8 mM NaH_2_PO_4_ and 22.2 mM KHPO_4_), and measurements OD of 412 nm were performed after 5, 10, 15, and 20 min. Chelex was used as negative control, 9,10-phenanthrenequinone (30 μM) as positive control, and the analysis was repeated with three replicates. DTT assay is a cell free assay which is based on the oxidation of DTT molecule and thus the linear rate of DTT loss. DTT-assay is most sensitive metals and quinones, with metals in PM usually dominating the DTT response (Charrier & Anastasio [13]. Rate of DTT loss is presented as μM/min^− 1^ per km^− 1^ and DTT tests were performed at Biosafe Biological Safety Solutions Ltd. in Kuopio.

### Cell culture

RAW264.7 mouse macrophages (ATCC, Rockville, MD, USA) were chosen as exposed cells in the present study as they have been used in multiple PM toxicity studies [28, 34, 64]. Moreover, macrophages are a major immune defence cell type in the lungs, which reacts towards particles in the lower airways. Therefore, they are a relevant model for the engine exhaust particles. Cells were cultured in RPMI culture medium with 10% heat inactivated fetal bovine serum containing 1% L-glutamine and 1% penicillin/streptomycin (all Gibco®, Life Technologies, CA, USA) in a humidified incubator at 37 °C and 5% CO_2_. Cells were seeded at 5 × 10^5^ cells/ml/well on 12-well plates (Corning Inc., NY, USA) and grown for 24 h. One hour prior to the PM exposure experiments, fresh culture medium was changed.

### Exposure experiments

Before the cell exposure, the PM samples were dispersed into 1 ml of pyrogen-free water (W1503, Sigma-Aldrich) with 6% of dimethyl sulfoxide (DMSO) (final concentration of DMSO in wells being 0.075 to 0.3% depending on the dose) by sonication (30 min, below 35 °C) in an ultrasonic water bath (FinnSonic M03, FinnSonic Ltd., Finland). This pre-treatment of PM was assessed to remove the possible PM aggregates and therefore ensure the proper dispersion of PM on cells. The PM doses corresponded to certain volumes of the diluted exhaust gas, which were 0.25 m^3^, 0.5 m^3^, and 1 m^3^ for Euro 6a cars and 0.01 m^3^, 0.025 m^3^, 0.05 m^3^, and 0.1 m^3^ for the Euro 2 car. Exposure method and doses were chosen based on experience and knowledge from previous studies and pilot experiments, thus, the volume-based doses correspond to mass-based doses used in our earlier studies [28, 29]. The rough estimations of PM masses for each volume-based dose in the present study were calculated (Supplementary material table 2. Additional file 1). PM samples were administered onto the cells followed by incubation of 24 h at 37 °C and 5% CO_2_. Experiments included untreated cells as controls and cells exposed with DMSO and pyrogen-free H_2_O as vehicle controls. Additionally, cells were exposed with blank filter samples to rule out any toxicological effects from the filter materials. For the different Euro 6 cars, four independent exposure experiments were conducted yielding *n* = 4. However, due to lack of PM samples, the independent exposure experiment number were only *n* = 2 for the DI-E2 PM dose of 0.01m^3^, and for the other DI-E2 doses total of three independent experiments were conducted resulting to *n* = 3. After the 24-h exposure, the cell culture medium was collected and frozen at − 80 °C for further cytokine analysis. The cells were rinsed with 1 ml of Dulbecco’s phosphate buffered saline (PBS) and cells were resuspended to PBS by scraping them from the bottom of the wells. Inflammatory markers, cellular metabolic activity (CMA), cell membrane integrity (CMI), cell viability and analysis of the reactive oxygen species (ROS) were analysed as described by [28]. Additionally, analysis mutagenicity was conducted as described further.

### Toxicological analyses

To rule out any potential interference of the PM in the samples with the measurements, 96-well plates containing corresponding doses of the PM without the cells were used for CMA and CMI analyses.

#### Analysis of inflammatory mediators

Levels of the pro-inflammatory markers tumor necrosis factor alpha (TNFα) and macrophage inflammatory protein 2 (MIP-2) were measured from the culture medium using commercially available enzyme linked immunosorbent assay (ELISA) kits (R&D-Systems, Minneapolis, MN, USA) on 96-well plates (Nunc Maxisorp, Invitrogen, USA) according to the manufacturer’s instructions. Lipopolysaccharide (LPS) was used as a positive control to ensure functionality of the method.

#### Analysis of cellular metabolic activity

Cellular metabolic activity of the RAW264.7 macrophages was detected with the 3-(4,5-dimethylthiazol-2-yl)-2,5-diphenyltetrazolium bromide (MTT)-assay on 96-well plates from two 100 μl aliquots of each exposed cell culture. The absorbance was detected with a plate reader photometrically (VICTOR3™ Multilabel Counter model 1420–051, PerkinElmer, USA and Synergy H1, BioTek, USA) at 570 nm. The results of the MTT-assay are presented as percentage of CMA compared to control cells.

#### Analysis of cell membrane integrity

CMI, which also indicates the viability of the cells, was analysed by propidium iodide (PI) exclusion assay that was assessed directly after DCF assay by adding PI solution (0.5 mg/ml (w/v) in ddH_2_O) on wells followed by incubation of 20 min at 37 °C and 5% CO_2_. Fluorescence was detected by plate reader (VICTOR3™ Multilabel Counter model 1420–051, PerkinElmer, USA and Synergy H1, BioTek, USA, excitation at 540 nm and emission at 610 nm) followed by a 20 μl addition of 10% (v/v, in ddH_2_O) Triton X-100 to lyse the cells. Plates were incubated on a plate shaker for 20 min in dark at room temperature, and the fluorescence measurement was repeated to detect maximum PI fluorescence.

#### Analysis of cell viability

Viability of the RAW264.7 cells were analysed using ChemoMetec Nucleocounter NC-3000™ (ChemoMetec A/S, Allerod, Denmark). Cells were stained with a solution 13 (ChemoMetec A/S), containing acridine orange (AO), which stains all the cells, and 4′,6′-diamidino-2-phenylindole (DAPI) which binds strongly to DNA but takes time to penetrate the cell membrane, thus dying only the dead cells at the moment of measurement. Analyses were performed using A8 slides (ChemoMetec A/S). As both CMI and cell viability measurements with Nucleocounter indicate the cell viability, henceforth, results of Nucleocounter measurement are called vitality measurements.

#### Analysis of intracellular oxidative stress

The intracellular oxidative stress was assessed using 2′,7′-dichlorofluorescein diacetate (H_2_DCF-DA) in DCF-assay on 96-well plates from two 100 μl aliquot of each exposure followed by addition of the H_2_DCF-DA into the wells. DCF-assay measures multiple kinds of intracellular oxidants and thus gives a general view of the oxidative stress level in the cells. Moreover, as oxidative stress was assessed with DCF in vitro, different metabolic products and their effect to oxidative stress affects the measurements oxidative stress here [14]. The fluorescence was detected with plate reader (VICTOR3™ Multilabel Counter model 1420–051, PerkinElmer, USA and Synergy H1, BioTek, USA, with excitation at 485 nm and emission at 530 nm) at three different time points (0 min, 30 min, 60 min) with incubation at 37 °C and 5% CO_2_ between the measurements. Area under curve (AUC) was calculated and used as the parameter for statistical analyses, with results presented as fold change compared to the control cells. Additionally, cells exposed with H_2_O_2_ were used as positive control.

#### Analysis of mutagenicity

Mutagenicity potential of the PM samples was analysed by microAmes reverse mutation test, which measures the mutations of histidine utilization capacity. In the present study, bacterial mutagenicity was compared to toxicological responses from cells. However, cell genotoxicity is relatively comparable to bacterial mutagenicity [41], with exception that bacterial mutagenicity test is also positive to compounds which necessarily are not direct DNA damaging agents [53]. MicroAmes test was conducted with *Salmonella typhimurium* strain TA98 with and without metabolic activation by rat liver fraction S9. Purpose of S9 rat liver fraction is to include the possible different metabolites and their potential effects to mutations. DMSO was used as vehicle control and 4-nitroquinoline 1-oxide (4-NQO) and 2-amino anthracene were used as positive controls without and with S9-mix, respectively. Briefly, the culture of tester strain TA98 was grown in Nutrient Broth #2 supplemented with 10 mg/ml ampicillin at + 37 °C for 16 h in shaking water bath. Thereafter, appropriate volumes of different samples were added with overnight culture of TA98 into minimum glucose medium with or without the S9 fraction on the 24-well plate which was incubated 2 h at + 37 °C. After incubation, contents of each well of the 24-well plate were divided into 48 wells on a 384-well plate, which was subsequently incubated in an airtight plastic bag for 72 h at + 37 °C. Mutations were detected by colour change of purple to yellow due to pH change caused by proliferation of the tester strain. Results are presented as k_rev_/km which indicates mutagenic activity by unit of thousands of revertant, caused by the PM emissions from driving distance of 1 km.

### Statistical analysis

The various responses from RAW264.7 cells to PM exposures were tested against the corresponding control cells with one-way Welch’s analysis of variance (ANOVA), which allows for unequal variances unlike traditional ANOVA yet performs similarly when variances are equal. Comparisons of sample means after a significant result in Welch’s ANOVA were performed by Dunnett’s T3 post-hoc test incorporating correction for multiple comparisons. All the differences were considered as statistically significant at *p* < 0.05. The data were analysed using the SPSS Statistics version 25.0 (SPSS Inc. Chicago, IL, USA).

## Results

### PM emissions and PM chemical composition

PM and nitrogen oxide emissions from the exhaust of each car are presented in Table 1 with the sum of particle-phase inorganic elements and organic constituents. Results of EC/OC and TC measurements are presented in the Table 2.

**Table 1:** PM mass, particle number (PN) and NO_x_ emissions, and concentrations of PM-bound organic and inorganic constituents from exhaust gases of one Euro 2 and four Euro 6 cars. < DL indicates the measurements in which the results were below the detection limits

	**DI-E2**	**CNG**	**E10**	**E85**	**DI-E6**
*PM emissions*					
PM mass (mg/km)	62.6	0.48	1.35	0.92	0.64
PN (#/km)	1.28 × 10^14^	6.63 × 10^10^	7.63 × 10^11^	7.49 × 10^11^	5.62 × 10^9^
*Nitrogen oxides*					
NO_x_ (mg/km)	1150	201	31	16	463
*Organic constituents*					
Sum of total PAHs (μg/km)	32.2	0.53	6.52	5.44	0.97
*Inorganic elements (mg/km)*					
SO_4_^2−^	0.125	< DL	< DL	< DL	< DL
NO_3_^−^	0.125	< DL	< DL	< DL	0.053
Cl^−^	0.063	< DL	< DL	< DL	< DL

**Table 2:** OC, EC, and TC measurements from one Euro 2 and two Euro 6 cars. DI-E2 values are averages from three phases during collection and Euro 6 are from one phase

	**DI-E2**	**E10**	**E85**
OC, %	35	50	31
EC, %	58	50	5
TC, %	93	100	32

**Fig. 1:**
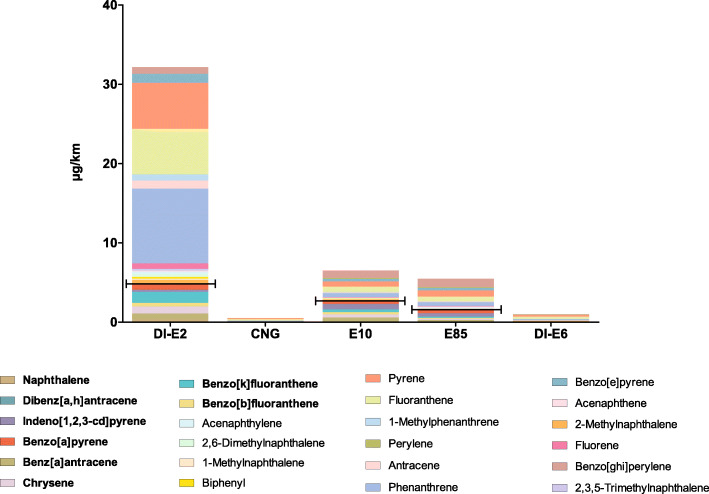
Masses of PM-bound polyaromatic hydrocarbons in diluted exhaust gas emitted during a driving distance of one kilometre (μg/km) from four different Euro 6 cars and one Euro 2 car. Carcinogenic PAHs are presented as bolded font and their sums as capped lines on figures

In the present study, total of 24 PAHs were analysed and presented in Fig. 1. Many of the measured PAHs have been categorised as carcinogenic by IARC. For example, PAHs such as naphthalene, benz [*a]* antracene, chrysene, benzo [*b*] fluoranthene, benzo [*k*] fluoranthene and indeno [1,2,3-*cd*] pyrene have been categorised by IARC to group 2B (possible carcinogenic to humans). Moreover, dibenzo [*a,h*] anthracene has been categorised to group 2A (Probably carcinogenic to humans) and benzo [*a*] pyrene to group 1 (carcinogenic to humans). Sums of these carcinogenic PAHs in different PM samples were DI-E2: 4.87 μg/km, CNG: 0.05 μg/km, E10: 2.76 μg/km, E85: 1.57 μg/km, DI-E6: 0.08 μg/km. The measured levels of benzo [*a*] pyrene were for DI-E2: 0.78 μg/km, E10: 0.47 μg/km, E85: 0.44 μg/km and DI-E6: 0.006 μg/km. No benzo [*a*] pyrene was detected in CNG PM samples.

#### Oxidative potential of the PM

**Fig. 2:**
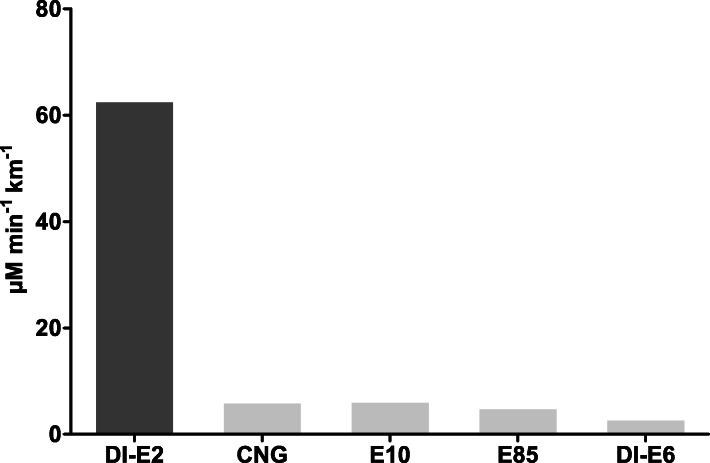
Oxidative potential measured with DTT assay from the PM samples of one Euro 2 and four Euro 6 cars. Results are shown as DTT rate of loss per km (μM/min^− 1^ km^− 1^). Results from Euro 2 car are marked with darker grey compared to the Euro 6 results

Oxidative potential results from DTT measurements are shown in Fig. 2. Highest oxidative potential was seen for DI-E2 originated PM with 62.4 μM/min^− 1^ km^− 1^ with oxidative potentials being below 6 μM/min^− 1^ km^− 1^ for PM samples originated from Euro 6 cars.

### Toxicological characteristics

Difference in the toxicological endpoints for blank filter, DMSO and H_2_O exposures were negligible compared to untreated control cells, thus, the untreated control cells are used as control in toxicological results.

#### Inflammatory mediators

**Fig. 3:**
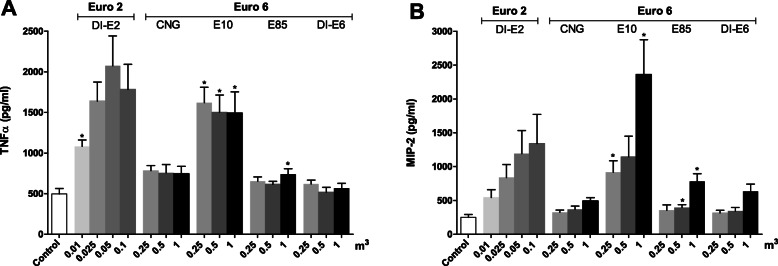
**a**. Levels of TNFα and **b**. MIP-2 (pg/ml) after a 24 h exposure of RAW 264.7 mouse macrophages to exhaust gas volume-based doses of PM samples from exhaust of one Euro 2 and four Euro 6 engines. The Euro 2 doses were one tenth of the doses used with Euro 6. Data reported as mean concentration with standard error of the mean (SEM). Asterisks indicate statistically significant differences from the control (Welch’s ANOVA and Dunnett’s t3 test; *p* < 0.05)

Levels of cytokine TNFα and MIP-2 secretion by RAW264.7 macrophages for all the different exhaust emission PM exposures were higher than in control cells (Fig. 3). For TNFα, the highest levels were measured from cells exposed to DI-E2 PM samples. Dose-dependent response was seen for the three lowest doses of DI-E2 PM with the lowest PM dose resulting to 221% (95% CI: 54.1, 257.3) and the second highest dose to 458% higher levels of TNFα (95% CI: 240.2, 675.0) compared to control cells. However, level of TNFα for the highest PM dose dropped close to level seen for the 0.025 m^3^ dose with 386% difference compared to control cells (95% CI: 220.4, 552.1) As for the Euro 6 cars, all doses of E10 exhaust PM exposure yielded clearly higher TNFα levels with the lowest dose resulting to 332% difference compared to the control cells (95% CI: 324.0, 413.5). The CNG engine exhaust PM samples showed higher levels of TNFα compared to control cells with the lowest PM dose resulting to 160% (95% CI: 119.3, 200.8) difference. The highest dose of E85 originated PM induced levels of TNFα with difference to control cells being 155% (95% CI: 125.0, 185.1) Moreover, the TNFα levels from exposures with DI-E6 PM were close to those measured from the control cells.

For MIP-2, the DI-E2 PM samples resulted with the highest PM dose to 587% (95% CI: 169.9, 1003.7) and with the lowest PM dose to 222% (95% CI: 115.3, 329.2) differences compared to control cells. Overall, the highest levels of MIP-2 were detected for the exposure of E10 PM samples with the highest PM dose resulting levels of MIP-2 up to 952% (95% CI: 846.5, 1057.2) and the lowest PM dose close to 369% (95% CI: 324.0, 413.5) differences, when compared to the control cells. Moreover, the highest PM dose for CNG, E85, and DI-E6 PM resulted to 202% (95% CI: 142.2, 260.8), 325% (95% CI: 221.4, 428.0) and 243% (95% CI: 122.2, 364.6) differences of MIP-2 levels compared to the control cells, whereas the lowest and middle doses resulted to only minor differences.

#### Cellular metabolic activity

**Fig. 4:**
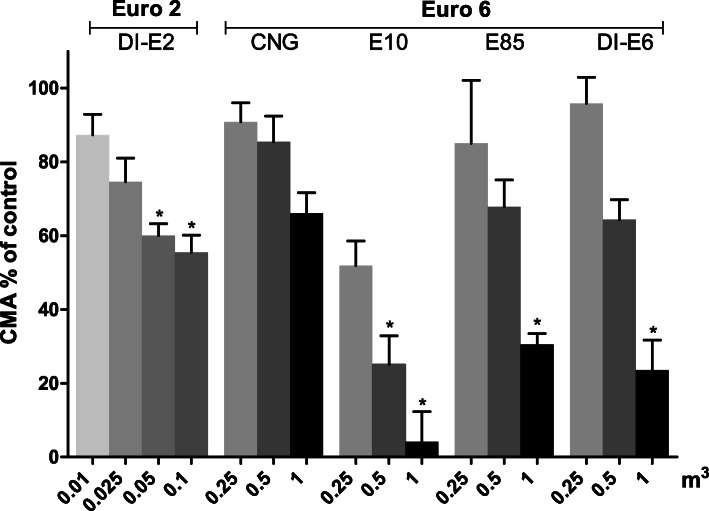
Decrease of CMA compared to control cells assessed with the MTT test after a 24 h exposure of RAW 264.7 mouse macrophages to exhaust gas volume-based doses of PM samples. The Euro 2 doses were one tenth of the doses used with Euro 6. Data reported as mean CMA compared to control cells with standard error of the mean (SEM). Asterisks indicate statistically significant differences from the control (Welch’s ANOVA and Dunnett’s t3 test; *p* < 0.05)

All exhaust gas PM exposures caused dose-dependent decreases of CMA compared to control cells as assessed with MTT-assay on RAW264.7 macrophages (Fig. 4). The lowest CMAs were detected for the E10 PM samples, with the exposure to the lowest PM dose resulting to 51% decrease (95% CI: 5.2, 96.8) and the highest dose to 95% (95% CI: 47.3, 143.7) decrease compared to control cells. For the E85 PM, the lowest dose resulted to 17% decrease (95% CI: − 29.5, 63.5) in CMA and the highest PM exposure dose to 71% decrease (95% CI: 20.5, 120.5) compared to control cells. DI-E6 PM resulted to CMA decrease of 78% (95% CI: 31.9, 123.6) for the highest PM dose exposure, whereas decrease with the lowest PM dose was negligible. Overall, the lowest decreases of CMAs from different exhaust gas PM sample exposures were measured for the CNG, as the two lowest doses resulted to negligible decrease and the highest dose to 33% (95% CI: − 47.3, 115.1) decrease compared to control cells. As for the DI-E2 PM, the two highest doses resulted to decreases of 43% (95% CI: − 46.4, 133.8) and 47% (95% CI: − 45.8, 139.8), respectively.

#### Cell membrane integrity and vitality

**Fig. 5:**
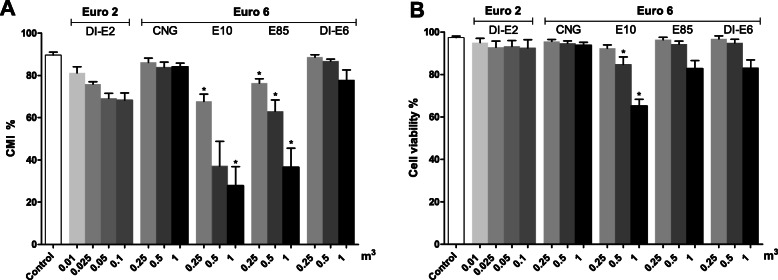
**a**. CMI detected with PI -exclusion method and **b**. cell vitality measurements after a 24 h exposure of RAW 264.7 mouse macrophages to exhaust gas volume-based doses of PM samples. The Euro 2 doses were one tenth of the doses used with Euro 6. Data reported as mean with standard error of the mean (SEM). Asterisks indicate statistically significant differences from the control (Welch’s ANOVA and Dunnett’s t3 test; *p* < 0.05)

Reduced CMI and cell vitality were detected on RAW264.7 macrophages exposed to exhaust PM samples (Fig. 5). The largest decrease of CMI were observed for the E10 PM exposures with the highest PM dose resulting to 27% (95% CI: − 0.8, 55.4) and the lowest dose to 66% CMI (95% CI: 53.6, 78.4). The lowest dose of E85 PM exposures resulted to 76% (95% CI: 69.3, 83.2) and the highest dose to 37% of CMI (95% CI: 7.1, 66.1) of CMI. Moreover, reduction in CMI with the CNG PM exposures were negligible compared to the control. As for the exhaust gas PM from the different diesel engines, the highest dose of DI-E6 PM resulted to 76% (95% CI: 62.3, 90.6) and the lowest dose to 88% of CMI (95% CI: 85.2, 91.4), whereas for the DI-E2 PM exposures, the CMI were 68% (95% CI: 29.1, 107.6) and 88% (95% CI: 81.1, 94.6), for largest and smallest PM doses, respectively.

Measurements of cell vitality displayed that E10, E85 and DI-E6 PM exposures caused dose-dependent decreases in viability compared to control cells. In contrast, for DI-E2 and CNG PM exposures, there were no considerable differences in viabilities between the different PM doses. E10 PM caused clear reduction of cell viability for doses of 0.5 m^3^ and 1 m^3^, with the highest dose resulting to 32% decrease (95% CI: 9.6, 54.5). Moreover, the highest doses of E85 and DI-E6 PM resulted close to 20% decreases of viability.

#### Intracellular oxidative stress

**Fig. 6:**
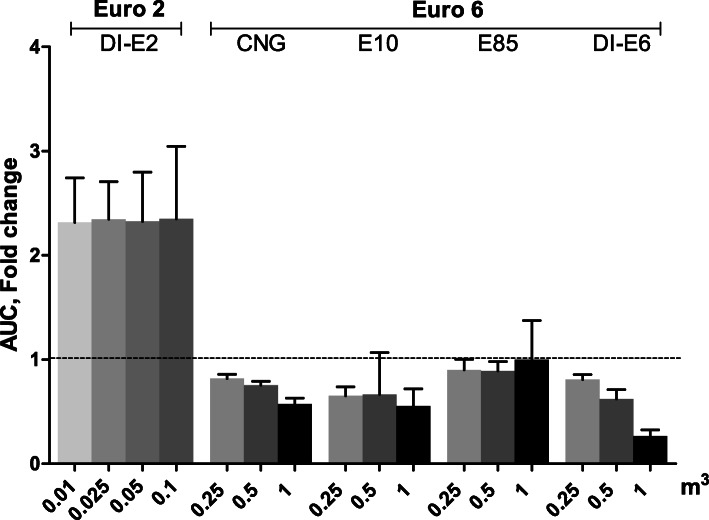
Oxidative stress assessed with DCF assay after a 24 h exposure of RAW 264.7 mouse macrophages to exhaust gas volume-based doses of PM samples. The Euro 2 doses were one tenth of the doses used with Euro 6. Data reported as mean fold change compared to control cells with standard error of the mean (SEM) and control level is indicated with a dashed line. Asterisks indicate statistically significant differences from the control (Welch’s ANOVA and Dunnett’s t3 test; *p* < 0.05)

The intracellular oxidative stress was only observed for the exposures of DI-E2 PM, with all the doses resulting up to two-fold differences compared to control cells. No intracellular oxidative stress above control cells were detected with different Euro 6 car PM samples (Fig. 6). Interestingly, the DI-E6 PM exposures resulted to dose-dependent decrease of the intracellular oxidative stress with the highest PM dose resulting to 72% decrease (95% CI: − 17.1, 217.1) compared to control cells.

#### Mutagenicity

**Fig. 7:**
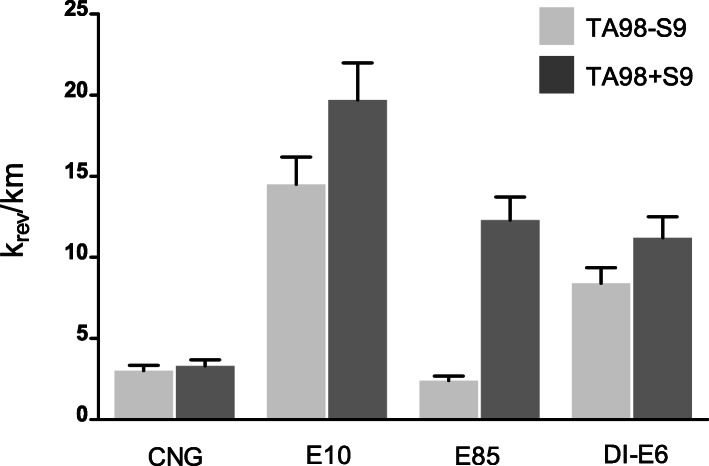
Mutagenicity of the four different Euro 6 cars originated PM measured with microAmes-assay. Results without S9 are marked with lighter grey whereas results with addition of S9 are marked with darker grey. Results are shown as mean with standard error of the mean (SEM) and are presented as k_rev_/km which indicates the mutagenicity potential of different Euro 6 car originated exhaust gas PM from driving distance of 1 km

Mutagenicity measurement with the microAmes test for different Euro 6 exhaust PM samples resulted to various levels of mutagenicity (Fig. 7). The highest mutagenic potential without addition of liver S9 was measured from E10 PM exposures with value of 14.5 k_rev_/km, followed by DI-E6 with 8.4 k_rev_/km, CNG with 3.0 k_rev_/km, and E85 with 2.4 k_rev_/km. With addition of liver piece S9, mutagenic potentials were overall higher and measured values were for E10: 19.7 k_rev_/km, DI-E6: 11.2 k_rev_/km, CNG: 3.3 k_rev_/km and E85 12.3 k_rev_/km.

## Discussion

Interestingly, as the new Euro 6 regulation for PN per km is 6 × 10^11^ [17], both gasoline Euro 6 regulated cars exceeded this. Yet, as this new regulation came in force in 2017, the standing PN regulation for cars in the present study was still the older one of 6 × 10^12^ #/km, which the gasoline cars in the present study complied with. Moreover, this high PN from gasoline cars might be due to the cold temperature (− 7 °C) during the testing phase, as cold temperature have been associated with increased PN and PAH concentrations from recent studies [52, 67]. The same might apply with the Euro 6 diesel car in association with NOx emissions. Additionally, the cold temperatures of the gasoline engines have also been observed to affect the concentrations of PAH emissions in the exhaust gases with increasing fashion [2, 32]. Interestingly, however not reported here, the CNG car emitted significant amount of methane, which is a more potent greenhouse gas compared to CO_2_.

Comparing the toxicity caused by chemical composition of the PM is not fully possible as the PM samples were collected from certain volume of exhaust gases from different cars and the concentrations of the PM in different samples were not entirely equal. However, as the difference between volumes of diluted exhaust gases per driving distance for Euro 6 cars were negligible, we can roughly compare the toxicological responses with the exhaust gas volume PM doses. Consequently, these results indicate the toxicity of the real-life PM emissions from cars fuelled with different fuels and equipped with different technologies. Different exhaust gas volume doses we used for the Euro 6 cars roughly correspond to the driving distances of 25 m, 50 m and 100 m. And as these were from diluted exhaust gas, the corresponding driving distances for raw exhaust gas were considerably lower. However, in real-life situation, the exhaust gases dilute very fast to surrounding environment. As for the Euro 2 car, the volume of diluted exhaust gas per driving distance were up to 30% higher and PM exposures doses one tenth, compared to the Euro 6 PM. However, the toxicological responses were equal or surpassed those from different Euro 6 car PM, indicating overall high PM emissions and toxicological potential from older diesel vehicles. In the following chapters, results from the toxicological measurements for different PM samples are discussed in more detail.

### DI-E2

In the present study, the Euro 2 car had the highest PM mass and PN emissions, which is coherent as the Euro 2 diesel car lacked any exhaust gas after treatment system. Additionally, the DI-E2 exhaust had the highest proportion of PM-bound carcinogenic PAH compounds which have been associated with toxicological responses in vitro in previous studies [28, 41, 73]. Furthermore, diesel exhaust particles (DEP) have demonstrated oxidative stress potential in previous studies whereas gasoline exhaust exposure have caused no increase of oxidative stress [11]. EC/OC analysis revealed that DI-E2 PM had 58% of EC which have been associated with oxidative potential and mutagenicity [41]. Moreover, the black carbon (BC), which is interrelated to EC, have been shown to induce overall high health effects as component of PM mixture [31]. Therefore, it is coherent that in the present study the DI-E2 PM showed the highest oxidative potential from DTT assay and in vitro exposure resulted in oxidative stress with DCF-assay even with the lowest PM dose, which however plateaued with other doses, indicating that even the low DI-E2 PM concentration results to high oxidative stress in vitro*.*

Measured concentrations from TNFα and MIP-2 indicate that the DI-E2 PM samples resulted in induced proinflammatory response in RAW264.7 macrophages, which is in line with previous studies where the emissions from diesel engine of older technology have displayed elevated inflammatory potential [28, 59]. Moreover, the observed dose response drop in TNFα results might be explained by that the highest PM dose exposure decreased functionality of the cells such a way that the production of the TNFα was compromised. This is supported by the results from MTT-assay as the highest dose of DI-E2 PM resulted to the lowest CMA compared to the lower doses of DI-E2 PM. However, similar drop of the MIP-2 secretion with the highest exposure dose were not observed.

As for the cell viability measurements, the CMI measurement showed slight decrease in cell viability with increased DI-E2 PM concentrations. However, such trend was not seen with cell viability from the vitality measurements, as overall the vitality measurements showed higher viability levels compared to the CMI measurement. Reason behind this may be that these measurements were performed later during the analysis day and at the time of measurement majority of the dead cells were destroyed such a way that there might have been nothing for dye solution to bind for. Results of the CMI are concordant with earlier studies as it has been observed that DEP induces decrease of cell viability [56]. Moreover, mutagenicity detected by microAmes test indicates that DI-E2 PM had high mutagenic potential, which supports the genotoxicity of DEP shown in earlier studies [24, 28].

### CNG

PM emissions from CNG powered engines have been shown to be remarkably lower than from gasoline and diesel fuelled engines, as composition of CNG fuel lacks carbon-carbon bonds, aromatic, and polyaromatic compounds [25]. Low PM emissions were observed in the present study, CNG exhaust containing the lowest mass concentrations of PM and second lowest PN emissions. PM emissions from CNG fuel combustion are likely originated from pyrolysis of hydrocarbons in lubrication oils, as speculated in prior studies [50].

Concerning the toxicological responses, CNG exhaust PM exposure yielded negligibly different responses for CMA, cell viabilities and oxidative stress measurements compared to control cells. Measurements from the inflammatory mediators showed that levels of TNFα were relatively higher, indicating inflammatory responses from cells induced by the CNG PM exposure, but not in a way that it would have compromised the metabolic activity or the viability of the cells. Mutagenicity results from microAmes test showed also low mutagenic potential. Similarly, low toxicity of the CNG exhaust PM has been observed in multiple previous studies [7, 49, 58].

### E10

Previous studies have shown the PM emissions from modern gasoline fleet have been observed to be higher compared to modern diesel fleet equipped with DPF [37, 45] which was also observed in the present study. However, the toxicity of the gasoline engine-originated PM have not been studied as extensively as toxicity of the DEP.

In the present study, the results from different toxicological endpoints were highest with E10 PM exposures compared to other Euro 6 PM sample exposures. Excessive toxicity caused by the E10 PM is coherent, since levels of PN, PM mass and PM-bound PAH compounds from exhaust were the highest among the Euro 6 regulated cars. Moreover, the EC/OC ratio showed that E10 PM had high levels of both fractions, and both fractions have been associated with toxicological responses [41]. Especially from the OC fraction, the carcinogenic PM-bound PAHs may play major role to toxicity, as levels of those were remarkably higher compared to the other Euro 6 PM samples. Similarly, to present study, PM originating from the gasoline exhaust have been shown to induce inflammatory responses in vitro in previous study of [70], where different exhaust PM from modern gasoline cars induced inflammation which was detected via increase of TNFα.

Subsequently, the highest decrease of CMA was additionally detected from the exposure of E10 exhaust PM samples indicating high cytotoxic effects to cells from gasoline originated PM. This have been also observed in previous study of [36], where aged gasoline PM was shown to induce cytotoxicity in vitro in primary and secondary cells. Based on the vitality and CMI measurements, we assume the cells were still alive even though their CMA was severely compromised. It indicates that the observed cytotoxicity was likely consequence from the ongoing process of apoptosis. The mutagenicity of E10 showed several times higher mutagenic potential compared to other Euro 6 car originated PM, thus indicating carcinogenicity, which have been associated with gasoline engine PM previously [63]. However, results from DCF and DTT assays indicate that exhaust PM from E10 fuelled passenger car did not result to oxidative stress in vitro nor had higher oxidative potential than other Euro 6 car originated PM.

### E85

The PM mass from the exhaust of E85 fuelled car were 30% lower than from E10 but the difference between PN numbers were negligible, indicating that E85 combustion produces overall smaller particles than E10. In fact, addition of ethanol to gasoline has been shown to affect the emissions; higher blends of ethanol have resulted in reduction of multiple different emissions components, especially the larger PM fraction [71]. Interesting remark is that EC/OC ratio for E85 was remarkably on side of OC. This indicates that major portion of PM might be originated from the lubrication oils, as OC fraction have been associated with lubrication oil in multiple studies [12, 51, 68]. This might also be one underlying reason for differences between toxicity of E10 and E85, as there are indications that carbonaceous core of PM work as important carrier of the more toxic compounds, e.g. PAHs [46]. Thus, the low levels of EC in E85 PM result that the cells do not get exposed in such a manner to most toxic compounds, compared to E10 PM where levels of EC were remarkably higher.

However, even though we saw no substantial differences between the PM mass and PN with E10 and E85 exhaust gases, the PM from two fuel types induced notably different toxicological responses. The inflammatory responses were considerably lower with E85 PM exposures compared to the E10 PM. Furthermore, results from cell viability measurements have similar trend, E85 causing lower loss of viability compared to E10 PM exposure. The highest toxicological response caused by exposure of E85 PM was loss of CMA, indicating that high doses of E85 PM induce cytotoxicity. Moreover, DTT and DCF assays indicate that exhaust PM from E85 fuelled passenger car had no notable acellular oxidative potential nor ability to induce intracellular oxidative stress. Indeed, previous studies have demonstrated that E85 PM has lower oxidative DNA damage potential and reduced concentrations of genotoxic PAH compounds compared to the gasoline exhaust PM [40]. Interestingly, mutagenicity assayed by microAmes test displayed multi-fold mutagenic potential when S9 was included in the assay. This suggests that the metabolic products of certain components in E85 PM induce higher mutagenicity.

### DI-E6

Of the different Euro 6 cars, PN concentration was the lowest in the DI-E6 exhaust with mass of PM and PM-bound PAH compounds being the second lowest. Ratio of PM mass and PN indicates that the size distribution of the emitted PM was shifted towards larger particles than seen in CNG exhaust. Low PM emissions from Euro 6 diesel car result largely from effective particle filtration by DPF, but the EGR systems have also been shown to lead to reduced PM emissions in exhaust gases [22, 43].

Most of the toxicological responses were negligible or insubstantial with the DI-E6 exhaust PM, as no oxidative stress, decrease of the cell viability nor secretion of inflammatory mediators were observed. However, CMA was reduced considerably with the highest dose of DI-E6 PM samples. Cell viability remained unchanged in the exposed cells, thus, indicating that the decrease of CMA was unrelated to decreased viability of the RAW264.7 cells. Similar decrease of the oxidative stress levels compared to control cells as seen in the present study, have been observed in previous studies after 24 h of the initial expose [73]. This was speculated to be result of the activation of intracellular antioxidants caused by high levels of oxidative stress at timepoint below 10 h after initial exposure. As in the present study, the toxicological analyses were conducted after 24 h of the initial PM exposure, thus, the intracellular oxidative stress levels below those of control cells might be explained by the transient oxidative stress and activation of the antioxidative responses. However, DI-E6 exhaust PM displayed very low oxidative potential in the DTT assay. This suggests that the results from DCF-assay are explained by other factor, for example the interference of the PM in the measurements which was not subtracted from the DCF-assay results. Moreover, considerably high mutagenic potential was observed from the DI-E6 PM compared to other toxicological responses, supporting the previously observed carcinogenicity of the DEP [24, 28]. Toxicity of the diesel emissions can be further decreased, as it has been shown in previous studies, that the emissions, especially the soot formation, can be effectively reduced by adding paraffinic fuel content. These fuels are also directly suitable for the current engines with no retrofit requirements and thus they would probably decrease the emissions even further [39, 44].

### Comparison of harmfulness of the emissions

Results of the present study show that the new emissions regulations for passenger cars have been efficient in reducing exhaust PM emissions. Toxicological results in the present study are in line with that, as Euro 2 diesel PM resulted to equal or elevated toxicity compared to the Euro 6 car PM exposures with substantially lower exhaust gas volume-based PM doses, thus, indicating multi-fold toxicity compared to PM emissions from the modern cars. Furthermore, to visually present comparison of results in the present study, we conducted table in which the toxicological results are numerically compared between different PM sample exposures and the Euro 2 results are extrapolated to dose of 1 m^3^/km (Supplementary material table 3. Additional file 1). This table illustrates the multi-fold difference in toxicity from Euro 2 exhaust PM compared to the Euro 6 PM exposures. However, authors note that this table is only at best, a crude extrapolation of the toxicological results. Overall, as the passenger car fleet can be up to 10 years old in the EU area (depending on country, [5]), legislation encouraging the replacement of aging cars with newer vehicles could be an effective way to decrease traffic-originated PM emissions.

As for the different Euro 6 cars, the modern diesel and CNG fuelled cars had the lowest PM emissions and levels PM-bound PAHs, which resulted to the lowest toxicological responses. Furthermore, the diesel engine exhaust PN concentrations are expected to decrease even further when selective catalytic reduction (SCR) catalyst is introduced [65]. However, the DI-E6 car had relative high NO_x_ emissions in the present study compared to the other Euro 6 cars. NO_x_ can contribute considerably to PM toxicity with process of initiating formation of nitro-PAHs, which possess high toxicological capacity [10]. Furthermore, even though in the present study, the observed NO_x_ emissions from modern diesel car were several times lower than that of older diesel car, some studies have reported as high levels of NO_x_ from Euro 6-regulated diesel cars as from the older diesel cars [15]. Additionally, the exhaust after-treatment system for NO_x_ have been shown to degrade over time [16]. Consequently, even though the PM emissions from new diesel cars are considerably low, the NO_x_ emissions remain a problem for the modern diesel cars. One solution for lower NO_x_ emissions could be the improved surveillance of tampering of the exhaust treatment systems as was shown by the “diesel scandal” at 2015 [62]. These steps have, indeed, been taken in the Europe lately as the recently implemented new laboratory test, Worldwide Harmonized Light Duty Test Produce (WLTP), is more representing for the real-life driving and emissions, compared to NEDC [57, 72]. Therefore, the WLTP with the addition of Real Driving Emissions (RDE) test, will subsequently pressure manufacturers to improve NO_x_ emission control methods.

According to the results of the present study, the PM emissions from CNG-powered car are the least toxic and thus CNG is a potent alternative fuel for passenger cars. Especially, if the CNG is made using bio-based instead fossil fuel-based gas, i.e. biomethane, which can utilize the existing CNG infrastructure and vehicle fleet, thus, resulting to lower greenhouse gas emissions [48]. However, as CNG is a gaseous form of fuel, some problems arise with properties required of cars, such as the reduced cargo space and lower range between refuelling [35, 66]. Furthermore, a recent study suggests that natural gas reservoirs and supply chains leak this powerful greenhouse gas with levels notably above those estimated earlier [9]. It was also seen that the methane emissions from the CNG powered car in this study were rather high [3].

Concerning the gasoline-fuelled cars, the present study indicates that that using high-concentration ethanol fuel in FFV reduces the PAH and PM mass concentrations, and toxicity of the exhaust PM, when compared to gasoline car. Similar results have been observed in the previous studies [40, 54, 71]. However, since the exhaust gas PN emissions from gasoline powered cars were considerably high compared to Euro 6 diesel and CNG-powered cars, one possibility for reducing PM emissions would be the implementation of the gasoline particle filters (GFP) which have been shown to reduce PM mass and PN emissions also in cold running temperatures [30] and decrease the toxicity of the gasoline exhaust [63]. Moreover, the multi-fold differences between the PAH concentrations in emissions from different Euro 6 cars is striking, as each of these cars are presentation of the modern technology.

Consequently, to comprehensive emission and toxicity results discussed here, we note that chemical and toxicological features of semi-volatile compounds’ emissions are almost unknown as they are not collected with gaseous or PM emissions [38]. Furthermore, it should be noted that the toxicological characteristics typically determined do not fully correspond the human exposure; e.g. the change of exhaust particle size distribution typical for older diesel vehicle [21] to size distributions typical for modern gasoline [33] and gas fuelled vehicles and engines [8] may significantly change the deposition of particles in human respiratory system [27] and thus also the exposure of people on toxicological features of particles.

## Conclusions

The present study indicates that exhaust PM from passenger cars using different fuels and technologies induces different toxicological responses in RAW264.7 macrophages exposed with an exhaust gas volume-based method. Main underlying reasons for this variability are probably the differences in the PM masses and PN in exhaust gases. However, differential chemical compositions of the PM, such as the EC/OC ratio and levels of PM-bound carcinogenic PAHs, likely factor in on the responses as well. Furthermore, the cold running temperature has most likely affected the emissions with increase in PM and PM-bound PAH concentrations, as such effects have been shown in previous studies. Overall, the PM emissions and toxicological responses from modern diesel and CNG powered cars were lowest in the present study. In contrast, the exhaust PM from gasoline and high-blend ethanol fuelled cars resulted to relatively high toxicological responses, with ethanol blend however decreasing the toxicological responses. Results of the older diesel car displayed that the new regulations to the diesel cars have effectively lowered the PM, PN and PM-bound PAH emissions in exhaust gases and the results from the toxicological measurements are consistent with that. Overall, it is striking that toxicological responses can be achieved with exhaust PM samples representing only such a short distance of driving when considering actual driving distances and the number of cars roaming the streets. Therefore, necessity of further regulations and research for passenger car exhaust gas emissions and their toxicity is evident.

## Supplementary information

**Additional file 1.**

## Data Availability

All data generated or analysed during this study are included in this published article and supplementary materials file.
