# Supplementary material for: Toxicological evaluation of exhaust emissions from light-duty vehicles using different fuel alternatives in sub-freezing conditions
Source: Part Fibre Toxicol. 2020 May 27;17:17. doi: 10.1186/s12989-020-00348-0 (PMC7251820; doi:10.1186/s12989-020-00348-0)
Supplement: Supplementary file 1 — Additional file 1. [file 12989_2020_348_MOESM1_ESM.docx]

**Supplementary material**

Details concerning the dilution of exhaust gases are presented in supplementary materials table 1., in which the dilution ratios (DR), volume of diluted exhaust gases (V_dil_) and volume of raw exhaust gases (V_raw_) are shown from different cars.

**Supplementary material Table 1**. Dilution rates, volumes of diluted exhaust gas and volumes of raw exhaust car from each car used in the present study.

|  | **DI-E2** | **CNG** | **E10** | **E85** | **DI-E6** |
| --- | --- | --- | --- | --- | --- |
| **DR** | 14.4 | 13.6 | 14.5 | 15.0 | 13.3 |
| **V_dil_** (m^3^/km) | 13.0 | 9.8 | 9.7 | 9.7 | 9.7 |
| **V_raw_** (m^3^/km) | 3.0 | 0.7 | 0.7 | 0.6 | 1.4 |

To compare our toxicological results to other studies, we have calculated the rough estimations how much mass of PM there were in each dose. Calculation was assessed by following equations (Supplementary material equation 1. and 2.). Results from calculations are presented as µg/dose in supplementary material table 2.

$\frac{Vdil (\frac{m3}{km})}{mg/km}=mg/m3$ (1)

$\frac{\frac{mg}{m3}}{dose}=\frac{mg}{dose} \to\mu g/dose$ (2)

**Supplementary material Table 2**. Mass (µg/dose) of PM in exposures doses from different cars used in the present study.

| **Dose** | **DI-E2** | **CNG** | **E10** | **E85** | **DI-E6** |
| --- | --- | --- | --- | --- | --- |
| 0.01 | 48 |  |  |  |  |
| 0.025/0.25 | 120 | 16 | 35 | 24 | 12 |
| 0.05/0.5 | 240 | 33 | 70 | 47 | 25 |
| 0.1/1 | 480 | 65 | 140 | 95 | 50 |

To present differences in toxicological responses from different PM exposures, we conducted a table in which toxicological results are presented as dose of 1 m^3^ per km, therefore taking into the account the driving distances. This is concluded by multiplying the toxicological result with the volume of diluted exhaust gas per km from each sample. After, the toxicological result from different PM exposures for each toxicological endpoint analyse, which had the lowest toxicological response, were set as number 1 and other results presented as a fold change to this. The number 1 was set in the most toxicological analyses to toxicological response from CNG PM exposure. Exceptions were the DCF assay, TNFα measurement and microAmes assay without S9, as in both DCF and TNFα the toxicological response for the DI-E6 PM exposure was set as 1 and in microAmes assay the toxicological response from the E85 PM exposure. Moreover, we had to extrapolate the toxicological results from the DI-E2 PM due lower exposure dose by multiplying the fold change ten times.

**Supplementary material Table 3**. Summarises our findings from different toxicological endpoints with extrapolating results from dose of 1 m^3^/km. The lowest toxicological result from each assay is set as number 1 (bolded in the table) and results from other PM samples are compared to that and displayed as a fold-change.

|  | **DI-E2** | **CNG** | **E10** | **E85** | **DI-E6** |
| --- | --- | --- | --- | --- | --- |
| TNFα | 42.3 | 1.3 | 2.6 | 1.3 | **1** |
| MIP | 36.5 | **1** | 4.7 | 1.6 | 1.2 |
| CMA | 16.7 | **1** | 15.8 | 2.2 | 2.8 |
| CMI | 16.5 | **1** | 3.0 | 2.3 | 1.1 |
| Cell viability | 13.6 | **1** | 1.4 | 1.1 | 2.1 |
| Oxidative stress | 119.5 | 2.2 | 2.1 | 3.8 | **1** |
| Mutagenicity (-S9/+S9) | - | 1.3/**1** | 6.0/5.9 | **1**/3.7 | 3.5/3.4 |
